# Balancing Early Detection and Overtreatment in Prostate Cancer: The Emerging Role of EpihTERT

**DOI:** 10.3390/cancers17233799

**Published:** 2025-11-27

**Authors:** Simeon Santourlidis, Marcos J. Araúzo-Bravo, Mohamed Hassan, Marcelo L. Bendhack

**Affiliations:** 1 Epigenetics Laboratory for Human Health and Longevity, Institute of Transplantation Diagnostics and Cell Therapeutics, Medical Faculty, Heinrich-Heine University Duesseldorf, 40225 Duesseldorf, Germany; 2 Group of Computational Biology and Systems Biomedicine, Biogipuzkoa Health Research Institute, 20014 San Sebastian, Spain; 3 Basque Foundation for Science, Ikerbasque, 48013 Bilbao, Spain; 4 Department of Cell Biology and Histology, Faculty of Medicine and Nursing, University of Basque Country (UPV/EHU), 48940 Leioa, Spain; 5 Institut National de la Santé et de la Recherche Médicale, University of Strasbourg, 67000 Strasbourg, France; 6 Department of Surgery, Tulane University School of Medicine, New Orleans, LA 70112, USA; 7 Department of Urology, Red Cross University Hospital, Positivo University, Curitiba 80030-200, Brazil; 8 Department of Urology, N. S. das Graças Hospital, Curitiba 80030-200, Brazil

**Keywords:** Acheron, active surveillance, DNA methylation, EpihTERT, focal therapy, liquid biopsy, *hTAPAS*, *hTERT*, overdiagnosis, prostate cancer, PSA screening, THOR

## Abstract

Prostate Cancer (PCa) screening using Prostate-Specific Antigen (PSA) has significantly improved early detection but has also led to substantial overdiagnosis and overtreatment, particularly of indolent tumors. While active surveillance and focal therapies have mitigated some harms, distinguishing aggressive from non-threatening disease remains a critical clinical challenge. Emerging evidence highlights the epigenetic regulation of the human Telomerase Reverse Transcriptase (*hTERT*) gene as a promising biomarker for risk stratification. Cancer-specific hypermethylation within the TERT Hypermethylated Oncological Region (THOR) and the broader CpG island termed “Acheron” correlates with *hTERT* reactivation, tumor progression, and adverse outcomes. Additionally, suppression of the long non-coding (lnc) RNA human TERT Antisense Promoter-Associated (*hTAPAS*) contributes to the derepression of *hTERT*, providing a mechanistic link between DNA methylation and telomerase activation. Collectively, these epigenetic signatures, referred to as EpihTERT, can be detected in tissue and liquid biopsies, offering non-invasive assessment of tumor aggressiveness. Integration of EpihTERT profiling into clinical practice may enhance early diagnosis, refine patient selection for intervention, and reduce unnecessary treatments, bridging the gap between overdiagnosis and timely identification of clinically significant disease. Prospective multicenter validation is warranted to establish EpihTERT as a robust, translational biomarker in PCa management.

## Simple Summary

Early testing for prostate cancer has helped identify the disease at an earlier stage, but it also leads to many findings of slow-growing tumors that would never cause harm. This often results in unnecessary treatments that can affect quality of life. To better distinguish dangerous tumors from harmless ones, new biological markers are needed. Our research focuses on changes in the chemical structure of the gene that controls the activity of telomerase, an enzyme that allows cancer cells to keep dividing. These specific changes, which we collectively call EpihTERT, occur in cancer and can be measured in tissue samples and in blood. By studying these patterns, we aim to provide a more precise way to identify which tumors are likely to grow and spread. This may help doctors avoid overtreatment while ensuring timely therapy for men with aggressive disease, improving care and supporting more informed clinical decisions.

## 1. Introduction

### Impact of PSA-Based Prostate Cancer Screening

The introduction of prostate-specific antigen (PSA) testing in the late 1980s revolutionized the detection of prostate cancer (PCa). While the primary aim was to reduce mortality, PSA screening also introduced a substantial risk of overdiagnosis and overtreatment. Overdiagnosis refers to the detection of PCa that would not have become clinically apparent within a patient’s lifetime. Early epidemiologic analyses highlighted this concern: Etzioni et al. (2002) reported that PSA screening “leads to substantial overdiagnosis of tumors that would never have become clinically apparent.” Analysis of the National Cancer Institute’s Surveillance, Epidemiology, and End Results (SEER) registry data from 1988 through 1998 revealed overdiagnosis rates of approximately 29% for white men and 44% for black men [1]. In contrast, McGregor et al. (1998), defining overdiagnosis as the detection of disease by screening that would not have caused PCa death, estimated overdiagnosis rates exceeding 80% [2].

Draisma et al. (2009) demonstrated that the number of indolent PCa diagnoses is inflated by lead time and early detection [3]. Here, the mean lead times—the intervals by which PSA screening advances PCa diagnoses—have been widely estimated to range from 3 to 12 years. These analyses showed that the lead time is linked to the overdiagnosis frequency, a fraction of screen-detected cancers that would not have been diagnosed in the absence of screening. They confirmed an overdiagnosis which ranged from 23% to 42% of all screen-detected cancers. The authors highlight that many overdiagnosed patients receive curative treatment (surgery or radiation therapy) for cancers that might have never become clinically significant, and this is associated with substantial morbidity and costs.

Randomized trials have, however, demonstrated reductions in PCa-specific mortality. In the European Randomized Study of Screening for Prostate Cancer (ERSPC), after 9 years of follow-up in 162,387 men aged 55–69 years, PSA-based screening reduced PCa mortality by 20% but was associated with substantial overdiagnosis [4]. To prevent one death from PCa, 1410 men (or 1068 men who underwent screening) had to be screened, with an additional 48 treated [4]. Adjustments for nonattendance and contamination indicated that PSA screening reduced mortality by up to 31% among men actually screened, though this benefit must be weighed against the degree of overdiagnosis and overtreatment [5,6]. A recent 23-year update of the ERSPC trial has further refined these estimates and confirmed the long-term benefit of PSA-based screening. The extended follow-up demonstrated a sustained reduction in prostate-cancer mortality, with a substantially improved harm–benefit ratio compared with earlier analyses [7]. The absolute mortality reduction increased over time, while the number needed to diagnose and treat to prevent one prostate-cancer death decreased markedly. Nevertheless, the updated data also reaffirm that PSA-based screening continues to be accompanied by considerable overdiagnosis, underscoring the persistent need for more specific biomarkers that can identify clinically significant disease while minimizing the detection of indolent tumors [7]. It has been emphasized that PSA should be interpreted alongside other predictive factors to identify men at risk for symptomatic or life-threatening cancer while minimizing detection of indolent disease.

## 2. Current Challenges in Prostate Cancer Management

### 2.1. Radical Treatment and the Problem of Overtreatment

Radical prostatectomy has significantly decreased PCa mortality and metastasis risk, particularly in younger or higher-risk patients, as demonstrated in the pre-PSA era Scandinavian Prostate Cancer Group Study Number 4 (SPCG-4) trial [8]. However, these results cannot be directly extrapolated to the PSA-screened population, in which low-risk cancers predominate. SPCG-4 also showed that some tumors deemed low-risk at diagnosis can be life-threatening if untreated, highlighting the need for careful risk stratification.

PSA screening has substantially increased PCa incidence due to overdiagnosis, although it has been associated with a reduction in metastatic disease incidence from ~28 to 11 per 100,000 men [9]. Welch and Albertsen argued that the conventional goal of maximizing cancer detection is insufficient; in an analysis of US incidence trends during the last 20 years, they estimated that more than a million additional men have been diagnosed and treated for PCa because of introduction of PSA screening [10]. The aim should be to detect clinically significant tumors while avoiding intervention in indolent disease. Radical interventions, such as surgery, increased correspondingly. The Prostate Cancer Intervention Versus Observation Trial (PIVOT) demonstrated that radical prostatectomy did not significantly improve survival over observation in men with low-risk, primarily PSA-detected cancers [11,12,13]. Early intervention may result in morbidity, including impacts on urinary, sexual, and physical function. Microsimulation analyses confirmed that modest survival benefits from PSA screening can be offset by quality-of-life losses due to overtreatment [14].

Overdiagnosis and overtreatment rates vary by age and screening intensity, with younger men disproportionately exposed to unnecessary interventions [1,3].

### 2.2. Active Surveillance as a Risk-Adapted Strategy

Active surveillance (AS) has emerged as the preferred management for men with low-risk, localized PCa. Long-term prospective data from Klotz et al. (2015) demonstrated very low prostate-cancer-specific mortality over 10–15 years in carefully selected patients [15]. Similarly, the ProtecT trial showed no significant difference in PCa-specific mortality among monitoring, surgery, and radiotherapy over 15 years, although metastasis rates were slightly higher under monitoring [16]. These findings support AS as a safe, and widely validated alternative to immediate radical treatment, reducing overtreatment while maintaining oncologic safety.

Patient selection is critical. AS is recommended for men with insignificant or low-risk prostate cancer:

Clinical stage T1c;

PSA density < 15 ng/mL;

Gleason-Score ≤ 6;

≤2 positive cores;

<50% cancer per core [17].

The Toronto cohort [15] and the Johns Hopkins cohort [18] confirmed the long-term safety of AS, showing metastasis-free survival rates of 99% at 10 years among adherent patients. International consensus supports AS to prevent overtreatment while maintaining quality of life [19,20]. Cooperberg et al. further highlighted that AS is feasible outside specialized centers, showing low disease progression when selection criteria are applied [20].

PSA testing has transformed PCa detection and outcomes, but overdiagnosis and overtreatment remain challenges. Active surveillance provides a structured approach to mitigate these harms, emphasizing risk stratification, rigorous monitoring, and timely intervention for progressive disease.

### 2.3. Focal Therapy: A Middle Ground

In this context, minimally invasive focal therapies, particularly high-intensity focused ultrasound (HIFU), have gained traction as a middle ground between radical treatment and surveillance. HIFU delivers precise thermal ablation to targeted tumor foci while sparing surrounding tissues, thereby reducing treatment-associated morbidity [21]. The use of Magnetic Resonance Imaging (MRI)-ultrasound fusion guidance has further improved patient selection and treatment accuracy, enhancing oncological outcomes in focal HIFU therapy [22].

Clinical studies demonstrate that focal HIFU achieves favorable short- to mid-term cancer control in men with localized disease while maintaining urinary continence and erectile function in the majority of patients [23,24]. This therapeutic balance directly addresses the overtreatment dilemma by avoiding whole-gland therapy for indolent tumors, yet still provides intervention for lesions with a higher likelihood of progression. Importantly, HIFU also shows promise in the salvage setting after failed radiation therapy, offering a minimally invasive alternative with acceptable oncologic outcomes [25,26].

Despite these advances, focal therapy is not without challenges. Long-term outcome data remain limited, and questions persist regarding the optimal criteria for patient selection and follow-up. Nevertheless, advances in imaging and biopsy strategies have strengthened the ability to accurately identify clinically significant lesions amenable to focal treatment. Furthermore, the incorporation of focal therapy into treatment algorithms may alleviate patient anxiety associated with active surveillance, offering an intermediate option that balances oncological control with quality-of-life preservation.

The current body of evidence suggests that focal HIFU and related minimally invasive strategies represent a valuable addition to the management spectrum of localized PCa. They provide an opportunity to move beyond the binary choice of surveillance versus radical therapy, thereby directly addressing the dual challenge of avoiding overtreatment while ensuring timely intervention for indolent tumors at risk of progression [21,23,27].

### 2.4. Limitations of Prostate Biopsy

Despite its pivotal role in the diagnostic pathway, prostate biopsy exhibits several critical limitations that compromise its accuracy, safety, and clinical utility.

First, conventional systematic transrectal ultrasound-guided (TRUS) biopsy suffers from substantial sampling error and limited sensitivity. The Prostate MRI Imaging Study (PROMIS) trial demonstrated that TRUS biopsy misses a significant proportion of clinically important PCa, while simultaneously detecting indolent tumors of little clinical relevance. In contrast, multiparametric MRI (mpMRI) combined with targeted biopsy improved detection of clinically significant cancers and reduced overdetection, highlighting the inadequacies of standard biopsy [28]. These findings were confirmed in the PRECISION trial, which showed that MRI-targeted biopsy identified more clinically significant cancers and fewer clinically insignificant cancers compared to TRUS biopsy, thereby directly exposing the diagnostic shortcomings of systematic biopsy [29]. A Cochrane meta-analysis further consolidated this evidence, concluding that MRI-based strategies achieve superior diagnostic performance, while systematic biopsy alone is prone to both false-negatives and overdiagnosis [30].

Second, biopsy-based grading frequently fails to reflect the true pathological extent of disease. A landmark analysis by Epstein and colleagues revealed that more than one-third of patients diagnosed with Gleason score 6 disease on biopsy were upgraded at prostatectomy, underscoring the limited reliability of biopsy for risk stratification and treatment decision-making [31].

Finally, the procedure is not without morbidity. A systematic review by Loeb et al. demonstrated rising rates of post-biopsy infectious complications, including sepsis, despite routine prophylaxis, in addition to bleeding and urinary adverse events [32]. These complications highlight the safety limitations of transrectal biopsy, which is still widely performed.

Taken together, these data illustrate that prostate biopsy is hampered by diagnostic inaccuracy, pathological misclassification, and non-negligible morbidity. This evidence strongly supports the integration of advanced imaging, improved biopsy strategies, and novel biomarkers to overcome the intrinsic weaknesses of traditional biopsy in prostate cancer management.

## 3. The Need for Better Biomarkers

### 3.1. Limitations of Current Diagnostic Tools

Even with refinements in management strategies, a critical challenge persists: distinguishing indolent tumors from those with lethal potential. PSA lacks specificity, and histopathology alone does not fully capture tumor biology.

These findings highlight the need for more precise molecular biomarkers that can guide diagnostic and therapeutic decision-making in prostate cancer. This unmet need provides the biological rationale for developing markers that directly reflect malignant transformation at the molecular level.

In parallel, emerging supramolecular cancer-therapy approaches—such as self-assembled coordination platforms that improve drug stability, tumor selectivity, and controlled release—illustrate how advanced therapeutic concepts complement the development of equally refined molecular biomarkers [33].

### 3.2. Telomerase and Epigenetic Regulation of hTERT

Telomerase, through its catalytic subunit *hTERT*, is indispensable for maintaining telomere length and enabling cellular immortality, a defining hallmark of cancer [34,35]. While *hTERT* expression is tightly silenced in normal somatic tissues, its reactivation is observed in the overwhelming majority of human malignancies, including PCa, and underpins uncontrolled proliferation and tumor progression [36,37]. This reactivation is primarily governed not by genetic alterations but by profound epigenetic remodeling of the *hTERT* locus [38,39].

Central to this regulation is DNA methylation of CpG-rich domains flanking the *hTERT* transcriptional start site. Among these, the TERT Hypermethylated Oncological Region (THOR) has emerged as a paradoxical regulatory hotspot: rather than repressing transcription, cancer-specific hypermethylation of THOR facilitates *hTERT* activation [40]. THOR hypermethylation has been documented across diverse malignancies—including prostate, bladder, breast, pancreatic, and brain cancers—where it correlates with higher tumor grade, invasiveness, and adverse clinical outcome [39,40,41]. For example, in a retrospective cohort study of prostate cancer, it was shown that THOR hypermethylation is associated with higher Gleason scores, invasiveness, and increased recurrence rates [39]. The 5-year biochemical progression-free survival rate (BPFS) was significantly reduced in hypermethylated tumors. Even in the prognostically difficult group (Gleason 6/7), THOR hypermethylation was able to predict the clinical outcome [39].

Detailed methylation mapping has revealed that hypermethylation at selected CpG dinucleotides within THOR correlates with epigenetic silencing of the antisense long non-coding RNA *hTAPAS* [42], a natural repressor of *hTERT* [43]. In this model, THOR methylation indirectly unleashes *hTERT* transcription by disabling *hTAPAS*-mediated inhibition [42].

Beyond THOR, a broader CpG island spanning ~2.8 kb of the *hTERT* promoter, designated “Acheron,” has been identified as the critical epigenetic zone for telomerase regulation [40]. Aberrant methylation signatures within this region are consistently observed in multiple cancer types and constitute a robust foundation for biomarker development [40]. Together, Acherons including THOR represent epigenetically plastic domains whose cancer-specific methylation patterns promise to distinguish malignant from benign tissue with high sensitivity and specificity.

The clinical implications are substantial. Detection of differential methylation states within THOR and Acheron—collectively referred to as EpihTERT—enables non-invasive assessment of telomerase deregulation in both tissue and liquid biopsies [42]. This approach enables actionable molecular insights for early diagnosis, prognostic assessment, and therapeutic monitoring [40,42]. Importantly, these epigenetic biomarkers capture the molecular essence of tumor biology that remains invisible to conventional PSA testing or histopathology, thereby bridging the critical gap between overdiagnosis of indolent lesions and timely identification of aggressive disease. Prospective multicenter studies are warranted to validate EpihTERT as a biomarker for clinical-decision-making.

### 3.3. Beyond Prostate Cancer: Pan-Cancer Relevance of EpihTERT

Although prostate cancer represents a clinically compelling model for studying the diagnostic and prognostic utility of EpihTERT signatures, similar patterns of *hTERT* hypermethylation have been reported across a broad spectrum of human malignancies. The CpG-dense regions THOR and Acheron exhibit a remarkably conserved methylation architecture among diverse epithelial and neuroectodermal tumors, underscoring their universal role in telomerase reactivation. In bladder cancer, Leão et al. demonstrated that hypermethylation of THOR was associated with higher *TERT* expression and higher-risk disease in nonmuscle invasive bladder cancers (NMIBC) [41]. Increased THOR hypermethylation doubled the risk of stage progression of NMIBC bringing additional prognostic value, while the absence of this epigenetic alteration participates in an indolent phenotype [41]. Comparable hypermethylation has also been documented in thyroid cancer. The authors reported that *TERT* promoter DNA methylation upregulates TERT expression and associates with poor clinical outcomes of papillary thyroid cancer (PTC), thus holding the potential to be a valuable prognostic marker for PTC risk stratification [44]. Furthermore, *hTERT* promoter methylation appears as a predictor of poor outcome in pancreatic cancer [45]. In breast cancer (BC) THOR is significantly hypermethylated in malignant breast tissue when compared to benign tissue, differentiating malignant tumor from normal tissue from the earliest stage of disease [46]. Cells previously demethylated on THOR did not develop a histologic cancer phenotype in in vivo assays. The authors conclude that THOR hypermethylation is an important epigenetic mark in breast tumorigenesis, representing a promising biomarker and therapeutic target in BC [46]. In paediatric brain tumours hypermethylation upstream of the transcription start site of *hTERT* gene is associated with tumour progression and poor prognosis [47]. The authors stated that this region is easy to amplify, and the assay to establish hypermethylation can be performed on most tissues in most clinical laboratories. Therefore this is a potentially accessible biomarker for various cancers [47].

In summary, this evidence demonstrates that cancer-specific methylation of THOR represents a convergent epigenetic hallmark of malignancy (Table 1).

From a translational perspective, the recurrence of EpihTERT signatures across multiple tumor entities provides a unique opportunity to develop pan-cancer screening and monitoring assays based on targeted methylation profiling. Recent liquid-biopsy approaches employing cell-free DNA (cfDNA) methylation panels have successfully integrated diverse gene associated CpG markers into multi-cancer early detection (MCED) frameworks [48].

Within such platforms, the inclusion of THOR/Acheron-specific probes has the potential to improve sensitivity, tissue-of-origin classification and prognosis. Consequently, EpihTERT methylation represents not only a promising biomarker for risk stratification in prostate cancer but also a mechanistically grounded, broadly applicable molecular signature of malignancy.

The epigenetic regulation of *hTERT* exemplifies how paradoxical DNA methylation dynamics and lncRNA-mediated feedback converge to sustain telomerase activity in cancer. Systematic profiling of these signatures—particularly within THOR, *hTAPAS*, and Acheron—offers a powerful and clinically translatable framework for the development of next-generation biomarkers in PCa and beyond.

**Table 1:** Pan-cancer evidence linking TERT-promoter hypermethylation with malignancy.

Cancer Type	Association with Malignancy/Aggressiveness	Reference
Prostate cancer	THOR hypermethylation associated with higher Gleason grade, invasiveness, recurrence, and reduced progression free survival.	Castelo-Branco et al., 2016, Oncotarget [39]
Bladder cancer (NMIBC)	THOR hypermethylation correlates with elevated TERT, higher-risk phenotype, progression, worse outcome.	Leão et al., 2019, Int J Cancer [41]
Papillary thyroid carcinoma (PTC)	Upstream TERT-promoter hypermethylation linked to high TERT, aggressive disease, shorter PFS.	Li et al., 2024, Front Oncol [44]
Pancreatic ductal adenocarcinoma (PDAC)	TERT-promoter hypermethylation drives higher TERT and aggressive histology, poor prognosis.	Kumari et al., 2009, Ann Surg Oncol [45]
Breast cancer	THOR hypermethylation enriched in malignant tissue; associated with progression and prognosis.	Apolónio et al., 2022, Clin Epigenetics [46]
Paediatric brain tumours	UTSS hypermethylation associated with malignant transformation and poor survival.	Castelo-Branco et al., 2013, Lancet Oncol [47]
Hepatocellular carcinoma (HCC)	UTSS hypermethylation enriched in HCC and linked to malignant phenotype.	Zhang et al., 2015, Clin Res Hepatol Gastroenterol [49]

## 4. Perspectives

Current PSA screening and histopathology for PCa face a persistent challenge: they often fail to differentiate between indolent tumors and those that are potentially lethal. This limitation has contributed to widespread issues of overdiagnosis and overtreatment, where men with slow-growing, non-lethal cancers may undergo unnecessary interventions that compromise quality of life without improving survival outcomes. To address this, there is a critical need for more accurate, biologically informed biomarkers that can guide clinical decisions more precisely. In this context, the epigenetic regulation of telomerase, particularly through *hTERT* presents a promising new frontier.

*hTERT* is a hallmark of malignant transformation and progression. In PCa, *hTERT* expression is associated with distinct epigenetic changes, offering a unique molecular signature that can distinguish cancerous from non-cancerous tissue. One key mechanism involves the methylation of specific CpG-rich sequences within the *hTERT* 5’-region, notably THOR and the broader “Acheron” domain. Contrary to classical understanding, cancer-specific hypermethylation in these regions does not suppress gene activity but instead facilitates *hTERT* transcription by silencing inhibitory elements, such as the lncRNA *hTAPAS*. These epigenetic modifications are tightly linked with tumor aggressiveness and adverse clinical outcomes, making them ideal candidates for biomarker development.

The ability to detect THOR and Acheron methylation patterns—collectively termed EpihTERT—in both tissue and liquid biopsies opens new avenues for early and non-invasive prostate cancer detection. Crucially, EpihTERT biomarkers can identify telomerase activation, a direct indicator of malignant potential, even when PSA levels are ambiguous or histological findings are inconclusive. By reflecting the underlying molecular biology of the tumor, these markers offer a higher level of diagnostic precision. This could significantly reduce the risk of overtreatment by allowing clinicians to distinguish patients with aggressive, treatment-worthy cancers from those with indolent disease who may be better managed with active surveillance. From a practical standpoint, we envisage EpihTERT as a complementary tool within existing PSA- and MRI-based diagnostic pathways rather than a stand-alone replacement. First, in men with elevated or rising PSA but equivocal overall risk estimates, a liquid-biopsy EpihTERT assay (e.g., in blood or urine-derived cell-free DNA) could be applied as a pre-biopsy triage test to enrich for patients harboring telomerase-active, clinically significant tumors. Second, in men undergoing MRI-targeted and systematic biopsy, EpihTERT profiling of tissue cores could help refine risk stratification within the gray zone of Gleason 6–7 disease, thereby supporting decisions between active surveillance, focal therapy, and radical treatment. Third, in the follow-up of patients on active surveillance or after local treatment (including focal high-intensity focused ultrasound or radiotherapy), serial EpihTERT measurements in liquid biopsy could serve as an early indicator of molecular progression or minimal residual disease, complementing PSA kinetics and interval MRI. Together, these use-cases outline a realistic integration of EpihTERT into contemporary clinical pathways that warrants prospective evaluation.

**Figure 1:**
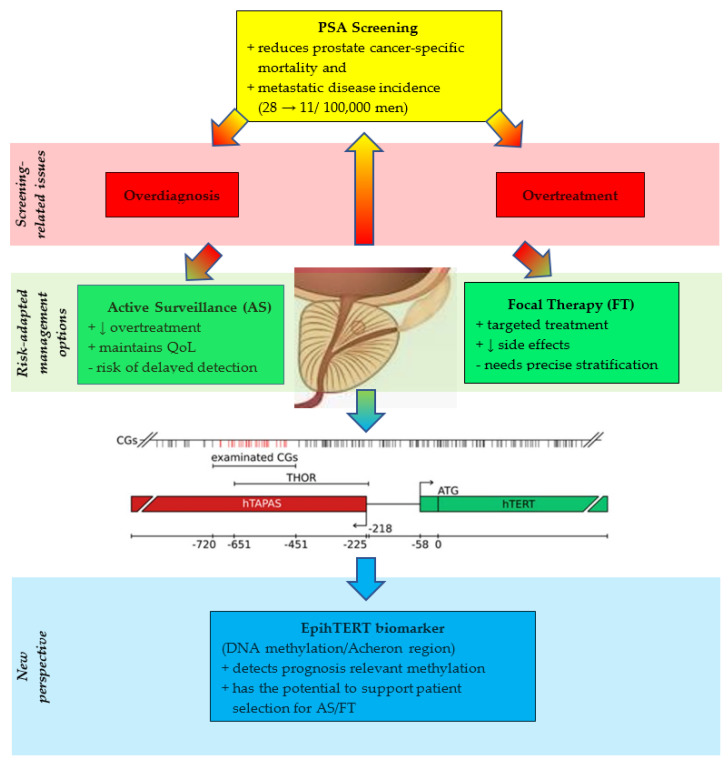
We are at the threshold of solving the PCa screening dilemma by elucidating the emerging role of EpihTERT. Overview of the proposed integration of the EpihTERT assay into contemporary prostate-cancer diagnostic pathways. Yellow, screening method. Red, screening-related issues. Green, risk-adapted management options. Blue, EpihTERT biomarker supplementing screening to reduce overdiagnosis, overtreatment and overtherapy using the exact location of the examined CpG rich region of a 215 bp 5′- fragment within THOR, ranging from −482 to −696 relative to the translational start site of *hTERT* gene as described in detail [40,42]. In men with elevated or rising PSA, liquid-biopsy EpihTERT testing may serve as a pre-biopsy triage tool to enrich for clinically significant, telomerase-active tumors. In patients undergoing MRI-targeted or systematic biopsy, tissue-based EpihTERT assessment can further refine risk stratification, particularly in the Gleason 6–7 range. Serial EpihTERT measurements may additionally support longitudinal monitoring during active surveillance or after local therapy. PSA, prostate specific antigene; QoL, quality of life.

In conclusion, telomerase regulation through epigenetic remodeling of *hTERT* provides a biologically meaningful, highly specific method for improving prostate cancer diagnosis and management. EpihTERT biomarkers represent a paradigm shift in cancer screening: moving from broad, non-specific markers like PSA to targeted, molecular-level diagnostics. This approach holds significant promise in minimizing the harms of overdiagnosis and overtreatment, thereby aligning therapeutic decisions more closely with individual disease biology. Prospective validation in large-scale studies will be essential, but the framework is already in place for a more precise and personalized era in prostate cancer care (Figure 1).

### Practical Considerations for Clinical Translation

The EpihTERT assay is based on highly specific methylation-specific PCR primer sets that were derived from precisely mapped, constitutively methylated CpG dinucleotides within the Acheron region. Because the assay relies on differential amplification efficiencies that are intrinsic to methylated versus unmethylated templates, its analytical performance is robust and inherently reproducible. Our experience indicates that this reproducibility is preserved across runs, sample types, and technical operators, and—given the simplicity and standardization of MSPCR—it is reasonable to expect comparable reproducibility across independent laboratories.

The assay uses standard molecular pathology equipment and requires only routine bisulfite conversion followed by real-time PCR. The workflow is rapid, inexpensive, and compatible with DNA isolated from formalin-fixed paraffin-embedded (FFPE) tissue, fresh tissue, urine-derived cell-free DNA and exosomal DNA. Thus, broad implementation in routine clinical diagnostics is technically straightforward and economically feasible.

In our diagnostic practice to date, we have not observed systematic false-positive or false-negative results. The assay’s specificity derives from the cancer-exclusive methylation patterns of the Acheron region. However, additional studies are warranted to determine whether methylation changes may occur in peritumoral histologically normal tissue, as suggested by the concept of “field effects”.

## 5. Conclusions

Early detection of prostate cancer has substantially reduced mortality but continues to suffer from overdiagnosis and overtreatment, underscoring the need for biomarkers capable of distinguishing indolent from clinically significant disease. In this study, we highlight the diagnostic potential of EpihTERT, a cancer-exclusive epigenetic signature located in the 5′ CpG island of the *hTERT* gene. Because telomerase activation is a fundamental hallmark of malignant transformation, EpihTERT provides a biologically grounded indicator of tumor aggressiveness that is absent in benign prostate tissue.

Our findings suggest that EpihTERT can complement current diagnostic tools by improving risk stratification across multiple steps of the clinical pathway. Potential applications include pre-biopsy triage in men with elevated PSA, refinement of risk categories in MRI-guided or systematic biopsies, and longitudinal molecular monitoring during active surveillance or after local therapy. Integrating EpihTERT into PSA- and MRI-based workflows may reduce unnecessary biopsies and treatments while enabling earlier identification of clinically significant tumors.

Future prospective clinical studies are warranted to validate these applications, determine optimal thresholds, and evaluate real-world clinical utility. If confirmed, EpihTERT has the potential to contribute meaningfully to individualized prostate cancer management and to support a more precise, biology-driven diagnostic strategy. Taken together, EpihTERT represents a biologically grounded, cancer-exclusive signature with genuine potential to reshape early detection, risk stratification, and longitudinal monitoring in prostate cancer and beyond.

## Data Availability

Not applicable.

## References

[1] Etzioni R., Penson D.F., Legler J.M., di Tommaso D., Boer R., Gann P.H., Feuer E.J. (2002). Overdiagnosis Due to Prostate-Specific Antigen Screening: Lessons from U.S. Prostate Cancer Incidence Trends. JNCI J. Natl. Cancer Inst..

[2] McGregor M., A Hanley J., Boivin J.F., McLean R.G. (1998). Screening for prostate cancer: Estimating the magnitude of overdetection. CMAJ.

[3] Draisma G., Etzioni R., Tsodikov A., Mariotto A., Wever E., Gulati R., Feuer E., de Koning H. (2009). Lead Time and Overdiagnosis in Prostate-Specific Antigen Screening: Importance of Methods and Context. JNCI J. Natl. Cancer Inst..

[4] Schröder F.H., Hugosson J., Roobol-Bouts M.J., Tammela T.L.J., Ciatto S., Nelen V., Kwiatkowski M., Lujan M., Lilja H., Zappa M. (2009). Screening and Prostate-Cancer Mortality in a Randomized European Study. N. Engl. J. Med..

[5] Roobol M.J., Kerkhof M., Schröder F.H., Cuzick J., Sasieni P., Hakama M., Stenman U.H., Ciatto S., Nelen V., Kwiatkowski M. (2009). Prostate Cancer Mortality Reduction by Prostate-Specific Antigen–Based Screening Adjusted for Nonattendance and Contamination in the European Randomised Study of Screening for Prostate Cancer (ERSPC). Eur. Urol..

[6] Roobol M.J., Carlsson S.V. (2013). Risk stratification in prostate cancer screening. Nat. Rev. Urol..

[7] Roobol M.J., de Vos I.I., Månsson M., Godtman R.A., Talala K.M., Hond E.D., Nelen V., Villers A., Poinas G., Kwiatkowski M. (2025). European Study of Prostate Cancer Screening — 23-Year Follow-up. N. Engl. J. Med..

[8] Bill-Axelson A., Holmberg L., Ruutu M., Garmo H., Stark J.R., Busch C., Nordling S., Häggman M., Andersson S.-O., Bratell S. (2011). Radical Prostatectomy versus Watchful Waiting in Early Prostate Cancer. N. Engl. J. Med..

[9] Welch H.G., Albertsen P.C. (2020). Reconsidering Prostate Cancer Mortality—The Future of PSA Screening. N. Engl. J. Med..

[10] Welch H.G., Albertsen P.C. (2009). Prostate Cancer Diagnosis and Treatment After the Introduction of Prostate-Specific Antigen Screening: 1986–2005. JNCI J. Natl. Cancer Inst..

[11] Wilt T.J., Brawer M.K., Barry M.J., Jones K.M., Kwon Y., Gingrich J.R., Aronson W.J., Nsouli I., Iyer P., Cartagena R. (2009). The Prostate cancer Intervention Versus Observation Trial:VA/NCI/AHRQ Cooperative Studies Program #407 (PIVOT): Design and baseline results of a randomized controlled trial comparing radical prostatectomy to watchful waiting for men with clinically localized prostate cancer. Contemp. Clin. Trials.

[12] Wilt T.J. (2014). Management of low risk and low PSA prostate cancer: Long term results from the prostate cancer intervention versus observation trial. Recent Results Cancer Res..

[13] Wilt T.J., Jones K.M., Barry M.J., Andriole G.L., Culkin D., Wheeler T., Aronson W.J., Brawer M.K. (2017). Follow-up of Prostatectomy versus Observation for Early Prostate Cancer. N. Engl. J. Med..

[14] Heijnsdijk E.A., Wever E.M., Auvinen A., Hugosson J., Ciatto S., Nelen V., Kwiatkowski M., Villers A., Páez A., Moss S.M. (2012). Quality-of-Life Effects of Prostate-Specific Antigen Screening. N. Engl. J. Med..

[15] Klotz L., Vesprini D., Sethukavalan P., Jethava V., Zhang L., Jain S., Yamamoto T., Mamedov A., Loblaw A. (2015). Long-Term Follow-Up of a Large Active Surveillance Cohort of Patients With Prostate Cancer. J. Clin. Oncol..

[16] Hamdy F.C., Donovan J.L., Lane J.A., Metcalfe C., Davis M., Turner E.L., Martin R.M., Young G.J., I Walsh E., Bryant R.J. (2023). Fifteen-Year Outcomes after Monitoring, Surgery, or Radiotherapy for Prostate Cancer. N. Engl. J. Med..

[17] Bastian P.J., Carter B.H., Bjartell A., Seitz M., Stanislaus P., Montorsi F., Stief C.G., Schröder F. (2009). Insignificant Prostate Cancer and Active Surveillance: From Definition to Clinical Implications. Eur. Urol..

[18] Tosoian J.J., Mamawala M., Epstein J.I., Landis P., Wolf S., Trock B.J., Carter H.B. (2015). Intermediate and Longer-Term Outcomes from a Prospective Active-Surveillance Program for Favorable-Risk Prostate Cancer. J. Clin. Oncol..

[19] van den Bergh R.C., Vasarainen H., van der Poel H.G., Vis-Maters J.J., Rietbergen J.B., Pickles T., Cornel E.B., Valdagni R., Jaspars J.J., van der Hoeven J. (2010). Short-term outcomes of the prospective multicentre ‘Prostate Cancer Research International: Active Surveillance’ study. BJU Int..

[20] Cooperberg M.R., Carroll P.R., Klotz L. (2011). Active Surveillance for Prostate Cancer: Progress and Promise. J. Clin. Oncol..

[21] Wu X., Wu Y., Ng C.F., Yee C.H., Chiu P.K. (2024). High-intensity focused ultrasound strategies for treating prostate cancer. Asian J. Androl..

[22] Yee C.-H., Chiu P.K.-F., Teoh J.Y.-C., Ng C.-F., Chan C.-K., Hou S.-M. (2021). High-Intensity Focused Ultrasound (HIFU) Focal Therapy for Localized Prostate Cancer with MRI-US Fusion Platform. Adv. Urol..

[23] Teoh J.Y.-C., Wong C.H.-M. (2025). When precision meets prostate cancer: The rising role of HIFU focal therapy. Prostate Cancer Prostatic Dis..

[24] Liu J., Feng Y.-G., Zhang C., Chen W.-Z. (2024). Advancements in high-intensity focused ultrasound for urological tumor research and application. Ann. Med. Surg..

[25] Crouzet S., Blana A., Murat F.J., Pasticier G., Brown S.C.W., Conti G.N., Ganzer R., Chapet O., Gelet A., Chaussy C.G. (2017). Salvage high-intensity focused ultrasound for locally recurrent prostate cancer after failed radiation therapy: Multi-institutional analysis of 418 patients. BJU Int..

[26] Sobhani S., Dadabhoy A., Ghoreifi A., Lebastchi A.H. (2024). Salvage High-Intensity Focused Ultrasound for Prostate Cancer after Radiation Failure: A Narrative Review. Curr. Oncol..

[27] Hopstaken J.S., Bomers J.G.R., Sedelaar M.J.P., Valerio M., Fütterer J.J., Rovers M.M. (2022). An updated systematic review on focal therapy in localized prostate cancer: What has changed over the past 5 years?. Eur. Urol..

[28] Ahmed H.U., El-Shater Bosaily A., Brown L.C., Gabe R., Kaplan R., Parmar M.K., Collaco-Moraes Y., Ward K., Hindley R.G., Freeman A. (2017). Diagnostic accuracy of multi-parametric MRI and TRUS biopsy in prostate cancer (PROMIS): A paired validating confirmatory study. Lancet.

[29] Kasivisvanathan V., Rannikko A.S., Borghi M., Panebianco V., Mynderse L.A., Vaarala M.H., Briganti A., Budäus L., Hellawell G., Hindley R.G. (2018). ECISION Study Group Collaborators. MRI-Targeted or Standard Biopsy for Prostate-Cancer Diagnosis. N. Engl. J. Med..

[30] Drost F.-J.H., Osses D.F., Nieboer D., Steyerberg E.W., Bangma C.H., Roobol M.J., Schoots I.G. (2019). Prostate MRI, with or without MRI-targeted biopsy, and systematic biopsy for detecting prostate cancer. Cochrane Database Syst. Rev..

[31] Epstein J.I., Feng Z., Trock B.J., Pierorazio P.M. (2012). Upgrading and downgrading of prostate cancer from biopsy to radical prostatectomy: Incidence and predictive factors using the modified Gleason grading system and factoring in tertiary grades. Eur. Urol..

[32] Loeb S., Vellekoop A., Ahmed H.U., Catto J., Emberton M., Nam R., Rosario D.J., Scattoni V., Lotan Y. (2013). Systematic Review of Complications of Prostate Biopsy. Eur. Urol..

[33] Dai Y., Sun J., Zhang X., Zhao J., Yang W., Zhou J., Gao Z., Wang Q., Yu F., Wang B. (2024). Supramolecular assembly boosting the phototherapy performances of BODIPYs. Co-ord. Chem. Rev..

[34] Shay J.W., Wright W.E. (2010). Telomeres and telomerase in normal and cancer stem cells. FEBS Lett..

[35] de Lange T. (2010). Telomere biology and DNA repair: Enemies with benefits. FEBS Lett..

[36] Kim N.W., Piatyszek M.A., Prowse K.R., Harley C.B., West M.D., Ho P.L.C., Coviello G.M., Wright W.E., Weinrich S.L., Shay J.W. (1994). Specific association of human telomerase activity with immortal cells and cancer. Science.

[37] Shay J.W., Wright W.E. (2011). Role of telomeres and telomerase in cancer. Semin. Cancer Biol..

[38] Guilleret I., Benhattar J. (2004). Unusual distribution of DNA methylation within the hTERT CpG island in tissues and cell lines. Biochem. Biophys. Res. Commun..

[39] Castelo-Branco P., Leão R., Lipman T., Campbell B., Lee D., Price A., Zhang C., Heidari A., Stephens D., Boerno S. (2016). A cancer-specific hypermethylation signature of the TERT promoter predicts biochemical relapse in prostate cancer: A retrospective cohort study. Oncotarget.

[40] Santourlidis S., Araúzo-Bravo M.J., Brodell R.T., Hassan M., Bendhack M.L. (2024). *hTERT* Epigenetics Provides New Perspectives for Diagnosis and Evidence-Based Guidance of Chemotherapy in Cancer. Int. J. Mol. Sci..

[41] Leão R., Lee D., Figueiredo A., Hermanns T., Wild P., Komosa M., Lau I., Mistry M., Nunes N.M., Price A.J. (2019). Combined genetic and epigenetic alterations of the TERT promoter affect clinical and biological behavior of bladder cancer. Int. J. Cancer.

[42] Ott P., Araúzo-Bravo M.J., Hoffmann M.J., Poyet C., Bendhack M.L., Santourlidis S., Erichsen L. (2022). Differential DNA Methylation of THOR and *hTAPAS* in the Regulation of *hTERT* and the Diagnosis of Cancer. Cancers.

[43] Malhotra S., Freeberg M.A., Winans S.J., Taylor J., Beemon K.L. (2017). A Novel Long Non-Coding RNA in the *hTERT* Promoter Region Regulates *hTERT* Expression. Non-Coding RNA.

[44] Li S., Xue J., Jiang K., Chen Y., Zhu L., Liu R. (2024). TERT promoter methylation is associated with high expression of TERT and poor prognosis in papillary thyroid cancer. Front. Oncol..

[45] Kumari A., Srinivasan R., Vasishta R.K., Wig J.D. (2009). Positive Regulation of Human Telomerase Reverse Transcriptase Gene Expression and Telomerase Activity by DNA Methylation in Pancreatic Cancer. Ann. Surg. Oncol..

[46] Apolónio J.D., Dias J.S., Fernandes M.T., Komosa M., Lipman T., Zhang C.H., Leão R., Lee D., Nunes N.M., Maia A.-T. (2022). THOR is a targetable epigenetic biomarker with clinical implications in breast cancer. Clin. Epigenet..

[47] Castelo-Branco P., Choufani S., Mack S., Gallagher D., Zhang C., Lipman T., Zhukova N., Walker E.J., Martin D., Merino D. (2013). Methylation of the TERT promoter and risk stratification of childhood brain tumours: An integrative genomic and molecular study. Lancet Oncol..

[48] Chen X., Gole J., Gore A., He Q., Lu M., Min J., Yuan Z., Yang X., Jiang Y., Zhang T. (2020). Non-invasive early detection of cancer four years before conventional diagnosis using a blood test. Nat. Commun..

[49] Zhang H., Weng X., Ye J., He L., Zhou D., Liu Y. (2015). Promoter hypermethylation of TERT is associated with hepatocellular carcinoma in the Han Chinese population. Clin. Res. Hepatol. Gastroenterol..

